# Life satisfaction and the risk of atherosclerotic cardiovascular disease in the general Japanese population: the Suita Study

**DOI:** 10.1265/ehpm.23-00125

**Published:** 2023-10-27

**Authors:** Ahmed Arafa, Rena Kashima, Yoshihiro Kokubo

**Affiliations:** 1 Department of Preventive Cardiology, National Cerebral and Cardiovascular Center, Suita, Japan; 2 Department of Public Health, Faculty of Medicine, Beni-Suef University, Beni-Suef, Egypt; 3 Department of Cardiovascular Pathophysiology and Therapeutics, Graduate School of Medicine, Osaka University, Suita, Japan

**Keywords:** Life satisfaction, Atherosclerotic cardiovascular diseases, Prospective cohort studies, Japan

## Abstract

**Background:**

Atherosclerotic cardiovascular disease (ASCVD) is a major cause of morbidity and mortality. Life satisfaction is a measure of mental health with a potential cardioprotective role. This study aimed to investigate the association between life satisfaction and ASCVD risk in the general Japanese population.

**Method:**

We used data from 6,877 people (30–84 years) registered in the Suita Study, a Japanese population-based prospective cohort study. All participants were free from stroke and coronary heart disease (CHD) at baseline. Then, participants were followed up for incident ASCVD, including cerebral infarction and CHD. Cox proportional hazards models were used to calculate the hazard ratio (HR) and 95% confidence interval (95% CI) of incident ASCVD according to life satisfaction.

**Results:**

Within 102,545 person-years (median follow-up = 16.6 years), 482 incident ASCVD events were identified. In the age- and sex-adjusted model, being very satisfied, rather satisfied, or not sure, compared to being dissatisfied with life, showed a lower risk of ASCVD: HR (95% CI) = 0.55 (0.41, 0.74), 0.67 (0.50, 0.89), and 0.57 (0.36, 0.88), respectively (p-trend < 0.001). The associations remained consistent after adjusting for stress and unfortunate events: HR (95% CI) = 0.57 (0.42, 0.77), 0.68 (0.50, 0.91), and 0.54 (0.35, 0.84), respectively (p-trend < 0.001). The results did not vary between cerebral infarction and CHD: HR (95% CI) for being very satisfied with life = 0.58 (0.37, 0.91) and 0.55 (0.36, 0.84), respectively.

**Conclusion:**

Life satisfaction was inversely associated with the risk of ASCVD in the investigated general Japanese population.

## 1. Introduction

Life satisfaction reflects physical and psychological well-being [1]. Unlike temporary emotions, life satisfaction is a stable cognitive judgment of essential life domains, such as health, work, and social life, making it a potential tool for investigating the collective influence of psychosocial attributes on health outcomes [1].

The previous decades have witnessed a decreasing age-standardized incidence of cardiovascular disease (CVD); however, the number of CVD cases increased, alongside the global population aging, from 31.3 million in 1990 to 55.5 million in 2019 [2]. Identifying modifiable risk factors for CVD is essential for risk prevention and early interventions [3].

Several modifiable psychosocial factors can influence the development of CVD [4]. Despite being shaped by several health and social factors, life satisfaction is modifiable through national policies promoting health determinants and individual interventions endorsing physical activity and other healthy practices [5, 6]. Life satisfaction is inversely associated with obesity and diabetes [7, 8]; both are major risk factors for CVD [9]. It is also associated with a lower Framingham Risk Score, an algorithm used to predict incident CVD events [10], suggesting that life satisfaction could protect from CVD.

The association between life satisfaction and CVD risk has been investigated, yet no decisive conclusions have been reached [11–17]. Most previous studies were limited by the short follow-up periods, suggesting that reverse causality bias might have influenced their results. In addition, evidence from Asian populations, who have distinctive perceptions of life satisfaction [18], is lacking.

In this context, we used data from the Suita Study, a Japanese prospective cohort study with a long follow-up period, to investigate the association between life satisfaction and atherosclerotic CVD (ASCVD) risk.

## 2. Methods

### 2.1. Participants

The study design of the Suita Study was previously described [19, 20]. In brief, the Suita population included two cohorts randomly selected by age and sex (recruited between 1989 and 1998) and a volunteer group (recruited between 1992 and 2006). A total of 8,360 eligible people from the urban city of Suita were included. Participants underwent a baseline health examination at the National Cerebral and Cardiovascular Center (NCVC) in Suita. First, they completed a questionnaire including information about their lifestyle and clinical history. Then, blood samples were drawn and a full clinical examination was conducted. Participants were asked to return every couple of years to the NCVC for follow-up. In this study, we excluded 1,483 participants for either having a positive history of stroke or coronary heart disease (CHD) (n = 367), lacking baseline data about life satisfaction (n = 667), or being lost to follow-up (n = 449). Eventually, 6,877 participants were included for analysis.

### 2.2. Outcome, exposure, and covariates

The health status of participants was examined during follow-ups in the form of medical exams every couple of years and questionnaires provided by mail or telephone annually. The medical records of participants were reviewed by registered physicians. ASCVD (outcome) was defined as incident cerebral infarction or CHD events diagnosed during the follow-up. CHD included myocardial infarction (MI), percutaneous coronary intervention, coronary artery bypass grafting, and sudden cardiac death. MI events were diagnosed based on the criteria of the WHO MONICA (World Health Organization Monitoring Trends and Determinants in Cardiovascular Disease) Project [21]. Cerebral infarction events were diagnosed based on the criteria of the US National Survey of Stroke [22].

Life satisfaction (exposure) was assessed using a question in the baseline questionnaire: *“Are you satisfied with your life as a whole?”* The responses were *“very satisfied”, “rather satisfied”, “dissatisfied”,* or *“not sure”*.

The covariates were collected from the baseline questionnaire, clinical examination, and blood samples. Hypertension was defined as systolic/diastolic blood pressure ≥ 140/90 mmHg or receiving medications, diabetes was defined as fasting blood glucose ≥ 126 mg/dL or receiving medications, chronic kidney disease (CKD) was defined as estimated glomerular filtration rate < 60 mL/min/1.73 m^2^, and hyperuricemia was defined as uric acid ≥ 7 mg/dL. Stress and experiencing unfortunate events were assessed by two questions: *“Is your daily life stressful?”* and *“Have you recently experienced unfortunate events?”* The possible responses for each question were *“yes”, “no”,* or *“not sure”*.

### 2.3. Statistical analysis

We applied the Chi-squared test to evaluate the differences in the baseline characteristics among different life satisfaction groups. Cox proportional hazards models were used to calculate hazard ratios (HR) and their 95% confidence intervals (95% CI) of ASCVD for participants who reported being very satisfied, rather satisfied, and not sure compared to those who reported being dissatisfied with life. Further, we stratified the results by all covariates. Person-years were calculated from the date of baseline assessment until the date of ASCVD event, death, leaving the study, or the end of follow-up (December 31, 2013), whichever came first. We adjusted the regression models for the following variables: age (continuous), sex, body mass index (BMI) (underweight, normal weight, overweight or obese), smoking behavior (non-current or current), alcohol consumption (non-current or current), physical activity (yes or no), sleep (<6, 6–7, >7 hours/night, or irregular), hypertension (yes or no), diabetes (yes or no), CKD (yes or no), hyperuricemia (yes or no), total cholesterol (TC) (<240 or ≥240 mg/dL), high-density lipoprotein-cholesterol (HDL-C) (<40 or ≥40 mg/dL), atrial fibrillation (AF), stress (yes, no, or not sure), and unfortunate events (yes, no, or not sure). SAS version 9.4 software (SAS Institute Inc, Cary, NC) was used for statistical analyses.

## 3. Results

This study included 6,877 participants (3,177 men and 3,700 women), aged 30–84 years (mean ± standard deviation = 54.7 ± 13.0 years). The groups of participants who reported being very satisfied, rather satisfied, or not sure, compared to those who were dissatisfied with life, included higher proportions of women and hypertension, but lower proportions of older age, smoking, alcohol consumption, hyperuricemia, stress, and experiencing unfortunate events (p-values < 0.05) (Table 1).

**Table 1:** Baseline characteristics of participants by their life satisfaction status (n = 6,877)

**Characteristics**	**Life satisfaction**				**P-difference**

	**Very satisfied**	**Rather satisfied**	**Not sure**	**Dissatisfied**	
Number of participants	2,881	2,756	458	782	—
Men, %	42.3	47.8	46.7	54.7	<0.001
≥65 years, %	35.0	21.0	20.7	13.2	<0.001
Body mass index ≥ 25 kg/m^2^, %	19.4	20.1	19.9	21.4	0.660
Current smokers, %	25.2	29.8	32.1	39.3	<0.001
Current drinkers, %	49.9	52.7	52.2	57.0	0.003
Sleep < 6 hours/night, %	26.5	32.6	39.1	36.6	<0.001
Physical activity, %	46.7	37.9	28.8	33.6	<0.001
Hypertension, %	34.7	29.4	28.4	25.1	<0.001
Diabetes, %	4.9	5.1	5.2	5.1	0.985
Chronic kidney disease, %	7.0	8.3	4.2	5.9	0.004
Hyperuricemia, %	8.7	11.3	10.9	11.0	0.010
High-density lipoprotein-cholesterol < 40 mg/dL, %	13.1	14.9	17.7	15.1	0.031
Total cholesterol ≥ 240 mg/dL, %	18.5	18.8	14.4	15.9	0.043
Atrial fibrillation, %	1.4	1.1	2.6	1.0	0.061
Stress, %	30.3	51.1	45.6	73.7	<0.001
Experiencing unfortunate events, %	21.9	26.7	29.0	36.6	<0.001

Chi-squared test.

Within a median follow-up period of 16.6 years (102,545 person-years), 482 incident ASCVD events were detected. The crude incidence of ASCVD/10,000 person-years was distributed by the life satisfaction category as follows: 46.7 for the very satisfied, 46.1 for the rather satisfied, 43.8 for the not sure, and 53.5 for the dissatisfied. In the age- and sex-adjusted model, being very satisfied, rather satisfied, or not sure was negatively associated with the risk of ASCVD: HR (95% CI) = 0.51 (0.38, 0.69), 0.66 (0.49, 0.87), and 0.64 (0.41, 0.98), respectively (p-trend < 0.001). The associations remained consistent after further adjustment for physical activity, sleep, BMI, smoking, alcohol consumption, hypertension, diabetes, CKD, hyperuricemia, AF, HDL-C, TC, stress, and unfortunate events: HR (95% CI) = 0.55 (0.41, 0.74), 0.67 (0.50, 0.89), and 0.57 (0.36, 0.88), respectively (p-trend < 0.001), and after adjusting for stress and unfortunate events: HR (95% CI) = 0.57 (0.42, 0.77), 0.68 (0.50, 0.91), and 0.54 (0.35, 0.84), respectively (p-trend < 0.001) (Table 2). The results did not materially vary by age, sex, or other ASCVD risk factors (p-interactions > 0.250) (Table 3). The associations between life satisfaction and each of cerebral infarction and CHD were similar: fully-adjusted HR (95% CI) for being very satisfied with life = 0.58 (0.37, 0.91) for cerebral infarction and 0.55 (0.36, 0.84) for CHD (Table 4).

**Table 2:** Life satisfaction and the risk of atherosclerotic cardiovascular disease (n = 6,877)

**Life satisfaction**	**Number of** ** incident cases**	**Incidence/10,000 ** **person-years**	**Model I** **HR (95% CI)**	**Model II** **HR (95% CI)**	**Model III** **HR (95% CI)**
Very satisfied	196	46.7	0.51 (0.38, 0.69)	0.55 (0.41, 0.74)	0.57 (0.42, 0.77)
Rather satisfied	194	46.1	0.66 (0.49, 0.87)	0.67 (0.50, 0.89)	0.68 (0.50, 0.91)
Not sure	30	43.8	0.64 (0.41, 0.98)	0.57 (0.36, 0.88)	0.54 (0.35, 0.84)
Dissatisfied	62	53.5	1 (Ref)	1 (Ref)	1 (Ref)
P-trend	—	—	<0.001	<0.001	<0.001

Cox-regression test

Model I: adjusted for age and sex

Model II: adjusted for age, sex, sleep, physical activity, BMI, smoking, alcohol consumption, hypertension, diabetes, CKD, hyperuricemia, AF, HDL-C, and TC

Model III: adjusted for age, sex, sleep, physical activity, BMI, smoking, alcohol consumption, hypertension, diabetes, CKD, hyperuricemia, AF, HDL-C, TC, stress, and unfortunate events

**Table 3:** Life satisfaction and the risk of atherosclerotic cardiovascular disease by risk factors (n = 6,877)

**Variables**		**Life satisfaction**				**P-interaction**

		**Very satisfied** **HR (95% CI)**	**Rather satisfied** **HR (95% CI)**	**Not sure** **HR (95% CI)**	**Dissatisfied**	
Age	<65 years	0.51 (0.35, 0.74)	0.58 (0.40, 0.83)	0.71 (0.43, 1.20)	1 (Ref)	0.873
	≥65 years	0.52 (0.32, 0.84)	0.74 (0.45, 1.21)	0.44 (0.19, 1.01)	1 (Ref)	
Sex	Men	0.51 (0.35, 0.73)	0.67 (0.48, 0.95)	0.69 (0.41, 1.16)	1 (Ref)	0.261
	Women	0.55 (0.33, 0.93)	0.66 (0.39, 1.12)	0.56 (0.25, 1.26)	1 (Ref)	
Body mass index	<25 kg/m^2^	0.51 (0.37, 0.72)	0.63 (0.46, 0.88)	0.67 (0.41, 1.10)	1 (Ref)	0.977
	≥25 kg/m^2^	0.49 (0.26, 0.92)	0.69 (0.38, 1.26)	0.50 (0.19, 1.32)	1 (Ref)	
Smoking	No	0.46 (0.32, 0.68)	0.61 (0.42, 0.89)	0.51 (0.28, 0.92)	1 (Ref)	0.660
	Yes	0.61 (0.39, 0.97)	0.76 (0.49, 1.19)	0.87 (0.45, 1.66)	1 (Ref)	
Alcohol consumption	No	0.59 (0.38, 0.91)	0.57 (0.37, 0.89)	0.71 (0.38, 1.31)	1 (Ref)	0.379
	Yes	0.45 (0.30, 0.67)	0.73 (0.50, 1.06)	0.56 (0.30, 1.04)	1 (Ref)	
Physical activity	No	0.59 (0.40, 0.86)	0.73 (0.50, 1.05)	0.66 (0.39, 1.13)	1 (Ref)	0.279
	Yes	0.43 (0.27, 0.68)	0.56 (0.35, 0.88)	0.63 (0.29, 1.34)	1 (Ref)	
Sleep	<6 hours/night	0.51 (0.29, 0.89)	0.74 (0.44, 1.24)	0.69 (0.32, 1.48)	1 (Ref)	0.847
	≥6 hours/night	0.49 (0.35, 0.70)	0.61 (0.43, 0.87)	0.61 (0.36, 1.04)	1 (Ref)	
Hypertension	No	0.49 (0.32, 0.76)	0.61 (0.40, 0.93)	0.96 (0.54, 1.71)	1 (Ref)	0.398
	Yes	0.54 (0.36, 0.80)	0.70 (0.47, 1.04)	0.40 (0.20, 0.79)	1 (Ref)	
Diabetes	No	0.49 (0.36, 0.67)	0.63 (0.46, 0.85)	0.66 (0.42, 1.05)	1 (Ref)	0.352
	Yes	0.74 (0.30, 1.86)	0.96 (0.39, 2.36)	0.40 (0.08, 2.00)	1 (Ref)	
Chronic kidney disease	No	0.50 (0.37, 0.68)	0.63 (0.46, 0.85)	0.66 (0.42, 1.03)	1 (Ref)	0.718
	Yes	0.66 (0.22, 2.00)	0.96 (0.33, 2.77)	—	1 (Ref)	
Hyperuricemia	No	0.50 (0.37, 0.69)	0.66 (0.48, 0.90)	0.60 (0.37, 0.98)	1 (Ref)	0.963
	Yes	0.56 (0.27, 1.17)	0.62 (0.30, 1.28)	0.77 (0.29, 2.07)	1 (Ref)	
High-density lipoprotein-cholesterol	≥40 mg/dL	0.45 (0.33, 0.63)	0.61 (0.44, 0.84)	0.51 (0.30, 0.87)	1 (Ref)	0.590
	<40 mg/dL	0.79 (0.41, 1.52)	0.84 (0.45, 1.60)	1.06 (0.47, 2.40)	1 (Ref)	
Total cholesterol	<240 mg/dL	0.53 (0.37, 0.74)	0.68 (0.48, 0.95)	0.63 (0.38, 1.04)	1 (Ref)	0.362
	≥240 mg/dL	0.50 (0.28, 0.88)	0.60 (0.35, 1.04)	0.73 (0.31, 1.76)	1 (Ref)	
Stress	No/not sure	0.50 (0.32, 0.80)	0.69 (0.44, 1.11)	0.71 (0.39, 1.29)	1 (Ref)	0.401
	Yes	0.54 (0.35, 0.82)	0.59 (0.40, 0.86)	0.48 (0.23, 1.03)	1 (Ref)	
Unfortunate events	No/not sure	0.58 (0.39, 0.86)	0.76 (0.52, 1.12)	0.93 (0.55, 1.57)	1 (Ref)	0.896
	Yes	0.48 (0.30, 0.76)	0.55 (0.35, 0.86)	0.26 (0.10, 0.68)	1 (Ref)	

Cox-regression test

Adjusted for age and sex

**Table 4:** Life satisfaction and the risk of cerebral infarction and coronary heart disease (n = 6,877)

**Life satisfaction**	**Cerebral infarction** **HR (95% CI)**	**Coronary heart disease** **HR (95% CI)**
Very satisfied	0.58 (0.37, 0.91)	0.55 (0.36, 0.84)
Rather satisfied	0.79 (0.52, 1.20)	0.64 (0.43, 0.96)
Not sure	0.58 (0.31, 1.10)	0.57 (0.31, 1.04)
Dissatisfied	1 (Ref)	1 (Ref)
P-trend	0.036	0.007

Cox-regression test

Adjusted for age, sex, sleep, physical activity, BMI, smoking, alcohol consumption, hypertension, diabetes, CKD, hyperuricemia, AF, HDL-C, TC, stress, and unfortunate events

## 4. Discussion

This study investigated whether life satisfaction influences the risk of ASCVD among Japanese people residing in Suita. Within a median follow-up period of 16.6 years, being very satisfied, rather satisfied, and not sure, compared to being dissatisfied with life, was associated with significant reductions in the risk of ASCVD. A dose-response relationship was observed with higher cardioprotective effects among those with higher satisfaction levels. These associations remained stable after adjusting for lifestyle, medical, and psychological variables, including stress.

The association between life satisfaction and CVD risk has been exclusively investigated in Western populations. In two studies from the British Whitehall II cohort, high life satisfaction was associated with a lower risk of CHD (average follow-up = 5.4 years): HR (95% CI) = 0.74 (0.55, 0.99), but not stroke/transient ischemic attack (average follow-up = 18 years): HR (95% CI) = 0.95 (0.72, 1.24) [8, 9]. The European Prospective Investigation into Cancer and Nutrition (EPIC)-Germany Study (average follow-up = 8 years) indicated that dissatisfaction with life was associated with the elevated risk of stroke in women, while the results in men did not reach statistical significance: HR (95% CI) = 1.69 (1.05, 2.73) and 1.40 (0.89, 2.19), respectively. However, no associations were observed with MI: HR (95% CI) = 1.13 (0.81, 1.58) in men and 1.26 (0.67, 2.38) in women [13]. A study using the Canadian Community Health Survey (CCHS) (average follow-up = 5.6 years) showed that dissatisfaction with life was significantly associated with the increased risk of CHD: HR (95% CI) = 1.46 (1.12, 1.92) [14]. The UK Biobank study (median follow-up = 11.5 years) showed that participants with lower life satisfaction posed increased risks of CVD, CHD, stroke, and heart failure: HR (95% CI) = 1.25 (1.18, 1.33), 1.26 (1.18, 1.35), 1.22 (1.07, 1.38), and 1.24 (1.09, 1.41), respectively [15]. The Copenhagen Aging and Midlife Biobank (CAMB) (maximum follow-up = 8 years) showed that every single standard deviation increase in the Satisfaction with Life Scale (SWLS) was associated with a lower risk of CHD: HR (95% CI) = 0.80 (0.72; 0.90) [16]. On the other hand, the US Health and Retirement Study (maximum follow-up = 10 years) indicated that life satisfaction was not associated with the risk of diabetes, hypertension, heart disease, or stroke [17].

The mechanisms linking life satisfaction to ASCVD risk are obscure, yet some explanations could be suggested. First, life satisfaction is associated with lower levels of chronic stress [23], a major risk factor for CVD [24]. However, adjusting for stress in this study did not affect the results. Second, life satisfaction has been linked to lower levels of inflammatory markers, such as C-reactive protein [25]. Chronic inflammation is associated with the development and progression of CVD [26]. Third, life satisfaction can influence health-related behaviors that could protect from CVD, such as engagement in physical and social activities and using preventive healthcare services [27–29]. Fourth, life satisfaction often correlates with having a strong social support network, including family, friends, and community. Social support plays a crucial role in cardiovascular health [4]. Fifth, sharing similar pathways could partly explain the influence of life satisfaction on ASCVD. Several chronic diseases that are linked to ASCVD could be a source of life dissatisfaction. However, when we adjusted our results for the traditional ASCVD risk factors, the results did not materially change.

One of the main strengths of this study is the prospective cohort design with the long follow-up period which allowed for a temporal association to be established and minimized the effect of reverse causality. Besides, the study was population-based and participants were randomly selected by age and sex to represent urban Japanese people. In addition, ASCVD and covariates were diagnosed using standard approaches. Furthermore, we adjusted our results for most ASCVD traditional risk factors, including stress, suggesting that the association between life satisfaction and ASCVD risk was not explained by confounders.

This study has some limitations that should be considered. First, life satisfaction was assessed as a whole using a simple question, and the domains of life satisfaction, such as work, health, and social life, were not examined separately. However, the Whitehall II cohort study showed that the protective effects of satisfaction with family life, job, and sex against CHD did not significantly vary [11]. Second, life satisfaction was assessed at baseline; therefore, its temporal stability could be questioned. However, a previous study showed that 76% of participants did not considerably change their life satisfaction level over 17 years [30]. Third, we had no data about dietary patterns, some sociodemographic characteristics, such as family structure, income, and occupation, and psychological factors, such as depression and anxiety; factors that could impact both life satisfaction [31] and ASCVD [32]. Fourth, the psychological factors that were included in the regression models were not assessed by validated psychological measures. Fifth, despite adjusting our results for most traditional ASCVD risk factors, it is still unlikely that their residual confounding effects were avoided due to the observational nature of this study and the complicated interactions between psychosocial factors and CVD.

## 5. Conclusion

We found that life satisfaction was inversely associated with the risk of ASCVD among the investigated Japanese people. The cardioprotective effect of life satisfaction was not explained by confounding variables, including traditional CVD risk factors and stress. Life satisfaction could be a target for policies aiming to enhance cardiovascular health. Future studies should explore the potential impacts of life satisfaction promotion interventions on reducing the risk of ASCVD.

## Abbreviations

AF: Atrial fibrillation
ASCVD: Atherosclerotic cardiovascular disease
BMI: Body mass index
CAMB: Copenhagen Aging and Midlife Biobank
CCHS: Canadian Community Health Survey
CHD: Coronary heart disease
CI: Confidence interval
CKD: Chronic kidney disease
EPIC: European Prospective Investigation into Cancer and Nutrition
HDL-C: High-density-lipoprotein-cholesterol
HR: Hazard ratio
MI: Myocardial infarction
NCVC: National Cerebral and Cardiovascular Center
SWLS: Satisfaction with Life Scale
TC: Total cholesterol
WHO MONICA: World Health Organization Monitoring Trends and Determinants in Cardiovascular Disease

## Declarations

### Availability of data and materials

Data will be provided by the corresponding author upon reasonable request.
